# Enhanced motor recovery in stroke patients through dual-modal intervention: motor imagery and task-oriented robot training

**DOI:** 10.1186/s12984-025-01831-5

**Published:** 2025-12-15

**Authors:** Jingyun Tan, Qing Yi, Xiaoping Meng, Haoyang Zhuge, Yu Qin, Haiquan Zhang, Yunsheng Zhang

**Affiliations:** 1 Shandong Sport University, Jinan, China; 2 Division of Sports Science and Physical Education, Tsinghua University, Beijing, China; 3 Institute of Physical Education and Social Sciences, Shandong Sports University, Jinan, China; 4 School of Sports Leisure, Shandong Sport University, Jinan, China; 5 Shandong Provincial Hospital Affiliated to Shandong First Medical University, Jinan, China; 6 Department of Rehabilitation Medicine, Shandong Provincial Third Hospital, Shandong University, Wuyingshan Road No. 16, Jinan, 250031 Shandong China

**Keywords:** fNIRS, Task-oriented robot training, Motor imagery, Stroke, Lower limb motor function

## Abstract

**Background:**

Motor imagery (MI) has garnered significant interest as a novel rehabilitation method for stroke. Additionally, task-oriented robot training has been shown to enhance lower limb motor function in patients with early-stage stroke. However, the therapeutic effects of combining these two approaches remain unclear, and the underlying mechanisms are not yet understood. This study aims to investigate the effects of MI combined with task-oriented robot training on the lower limb motor function of post-stroke patients.

**Methods:**

First-ever stroke patients meeting the inclusion criteria were recruited and randomly allocated eligible participants to the control group (*n* = 91) or the experimental group (*n* = 91). Based on routine conventional physical therapy, the experimental group received task-oriented robot training combined with MI training, whereas the control group received task-oriented robot training combined with muscle relaxation training. The outcome indicators are the Fugl-Meyer Assessment of Lower Extremity (FMA-LE), Berg Balance Scale (BBS), and spatio-temporal gait parameters, which reflect the patients’ lower limb motor function. The functional connectivity between regions is measured by functional near-infrared spectroscopy (fNIRS).

**Results:**

Significant improvements in FMA-LE and BBS were observed in the experimental group compared with the control group (*p* < 0.05). Although no significant differences were observed between groups post-treatment (*p* > 0.05), both groups demonstrated improved step frequency and gait speed scores and reduced gait cycle scores following intervention (*p* < 0.05). In addition, the experimental group showed significantly enhanced functional connectivity between the prefrontal cortex and motor-related regions compared to the control group (*p* < 0.05).

**Conclusions:**

Combining MI training with task-oriented robotic training can enhance lower limb motor function and enhance the brain’s functional connectivity. Changes in functional connectivity within the prefrontal cortex (PFC) and motor-related cortex may serve as a potential therapeutic target for promoting motor recovery in stroke patients. Future studies should incorporate task-based functional Magnetic Resonance Imaging (fMRI) data to elucidate the directionality of information flow between these brain regions, thereby advancing our understanding of causal interactions underlying functional improvements in post-stroke gait rehabilitation.

*Trial registration*: It was retrospectively registered at the Chinese Clinical Trial Registry on 8 July 2025 (Registration No. ChiCTR2500105631).

## Introduction

Stroke continues to be the second leading cause of death worldwide [1]. More than 70% of stroke survivors experience some level of physiological dysfunction, which can include motor, sensory, cognitive, and psychological disorders [2, 3]. Lower-limb motor dyskinesia is the most prevalent condition among stroke patients and can persist for years, leading to various adverse outcomes [4, 5]. These outcomes include increased vulnerability, functional dependence, difficulties in daily activities, and fatigue [6]. Compared to healthy individuals, stroke patients face a heightened risk of falling and take significantly longer to perform simple tasks such as turning and sitting down [7, 8]. Lower limb motor dysfunction is common in those recovering from a stroke, severely limiting their activity levels, increasing fall risks, and negatively impacting their ability to carry out daily tasks and engage socially [9]. Thus, restoring motor function in the lower limbs has become a priority in stroke rehabilitation. Task-oriented robotic training, which delivers high-intensity, repetitive, and body weight-supported gait exercises, has been widely recognized as an effective method for improving lower limb motor function after stroke [10, 11]. Task-oriented robotic training for lower limb rehabilitation has demonstrated efficacy in facilitating functional reorganization following central nervous system injury by delivering precise, repetitive movements that reinforce the acquisition and retention of correct motor patterns [12]. Research indicates that, in comparison to conventional methods, task-oriented robotic training may better facilitate the development of specific motor skills through task decomposition, thereby enhancing lower limb coordination and promoting gait symmetry [13]. Nevertheless, task-oriented robotic training predominantly facilitates peripheral neuromuscular adaptive changes, exerting a limited influence on central neural circuit reorganization [14].

In contrast, motor imagery (MI) demonstrates more pronounced effects on central neural modulation. MI involves the cognitive rehearsal of movements without physical execution, serving as a mental representation of limb actions [15]. Studies suggest that during motor evocation, MI activates cortical motor networks that overlap with those engaged in actual motor execution [16, 17]. Neuroimaging research further indicates that MI influences the premotor cortex, supplementary motor area, and primary sensorimotor cortex, potentially facilitating neural plasticity and remodeling [18, 19]. These areas are essential for regulating motor control and error correction, relying on interactions with the cerebellum and basal ganglia, and communicating with pathways such as the corticospinal tracts that relay motor commands to the spinal cord [20, 21]. Recent systematic reviews support the efficacy of MI as a beneficial adjunct therapy for upper limb rehabilitation [22].

Despite the promising potential of task-oriented robotic training and MI in post-stroke rehabilitation, several critical limitations persist. Firstly, the precise neural mechanisms by which these interventions facilitate brain functional reorganization remain incompletely understood, particularly due to a lack of comprehensive quantitative analyses addressing the dynamic changes in brain function [23, 24]. Secondly, there is a notable scarcity of large-scale randomized controlled trials evaluating whether MI can significantly augment the therapeutic effects of robotic training [25, 26]. Moreover, the role of interregional neural reorganization as a key mediator in lower limb motor function recovery has yet to be definitively established [27, 28]. These gaps presently constrain the advancement of integrated rehabilitation strategies that combine mechanical assistance with central nervous system modulation.

To definitively elucidate the neural mechanisms underpinning the synergistic effects of combined rehabilitation strategies, a reliable neuroimaging modality capable of capturing cortical network dynamics in an ecologically valid, movement-compatible context is required. Functional near-infrared spectroscopy (fNIRS) serves this purpose by offering a non-invasive, portable, and motion-tolerant method for monitoring cortical hemodynamics [29]. Due to these advantages, fNIRS has been extensively utilized in stroke populations to explore neuroplastic changes during motor rehabilitation [30]. In addition to traditional activation mapping, fNIRS facilitates more advanced analyses, such as functional connectivity, based on the temporal correlations of oxygenated hemoglobin (HbO_2_) signals, to evaluate the strength of communication between cortical regions [31]. Moreover, effective connectivity analysis can provide insights into the directional influences among brain areas, while graph theory approaches enable the quantification of global network properties like efficiency and modularity [32]. Collectively, these analytical capabilities position fNIRS as a powerful tool for characterizing the neural network reorganization that underpins functional recovery.

This study builds on existing insights by using fNIRS in a carefully designed randomized controlled trial (RCT) to explore the effects of combining MI with task-oriented robotic training on lower-limb functional recovery and cortical network plasticity in early post-stroke patients. We hypothesize that incorporating MI as an adjunctive intervention will optimize motor-related functional connectivity, thereby enhancing the effectiveness of robotic training and providing mechanistic evidence for the integration of peripheral mechanical assistance with central neural modulation.

## Method

### Participants

Between February 2023 and December 2023, 196 stroke patients were recruited through a convenience sampling method from the Department of Rehabilitation and Medicine at the Third Shandong Provincial Hospital. Participants were eligible for inclusion if they met the following criteria: (1) a diagnosis of stroke according to the International Cerebrovascular Disease Symposium criteria [33], confirmed by imaging modalities such as Computed Tomography (CT) or Magnetic Resonance Imaging (MRI), (2) initial onset of cerebral hemorrhage or cerebral infarction with a duration between one week and six months, (3) sufficient cognitive level and communication skills to engage in mental practice. Taken into account were the clinical judgment of the treating therapist, support from family, and the score on the Mini-Mental State Examination (MMSE) score of 24 or higher [34], (4) a Fugl-Meyer Assessment of Lower Extremity (FMA-LE) score below 34, (5) absence of skin damage around the hip, knee, and ankle, (6) a score of 25 or higher on the Kinesthetic and Visual Imagery Questionnaire-10 (KVIQ-10) [35], (7) a score of less than 24 points on the Hamilton Depression Scale [36], and (8) age between 25 and 75 years. Participants were excluded if they met any of the following criteria: (1) an unstable medical condition, (2) had conditions such as rheumatic diseases or dementia before stroke onset that could lead to persistent premorbid disability [37], (3) significant coordination dysfunction, (4) the presence of metal implants in the head or extensive scalp skin lesions, and (5) current treatment with antiepileptic or antidepressant medications.

This study was approved by the Research Ethics Boards at the Shandong Provincial Third Hospital (KYLL-2021002) and was registered retrospectively in the Chinese Clinical Trial Registry on 8 July 2025 (Registration No.: ChiCTR2500105631). All participants provided written informed consent before enrollment. This study initially began as an exploratory, preliminary trial with a small sample in 2022. Based on the preliminary results, we optimized the intervention protocol, for example, adjusting MI training duration and robot parameters, between December 2022 and January 2023, before launching the formal RCT in 2023. We registered in July 2025 to meet academic transparency standards. This study follows the CONSORT and SPIRIT recommendations [38].

### Sample size

A power analysis was conducted using G*Power software to determine the minimum sample size required to detect effects in an independent sample t-test. The calculation was based on the FMA-LE. The presumed effect size (Cohen’s d = 0.444) was derived from one study by Oos et al. [39] involving 44 participants (n_MIt_ = 21, n_MR_ = 23), where the significant difference of FMA-LE score yielded a value of 1, which was used to estimate the effect size. We used a standard deviation of 2.25 [40]. With a type I error of 0.05 and a power of 0.80, the estimated sample size was 81 participants per group. To account for a potential dropout rate of 20%, an additional 34 participants were recruited, yielding a final sample size of 196.

### Randomization

**Fig. 1:**
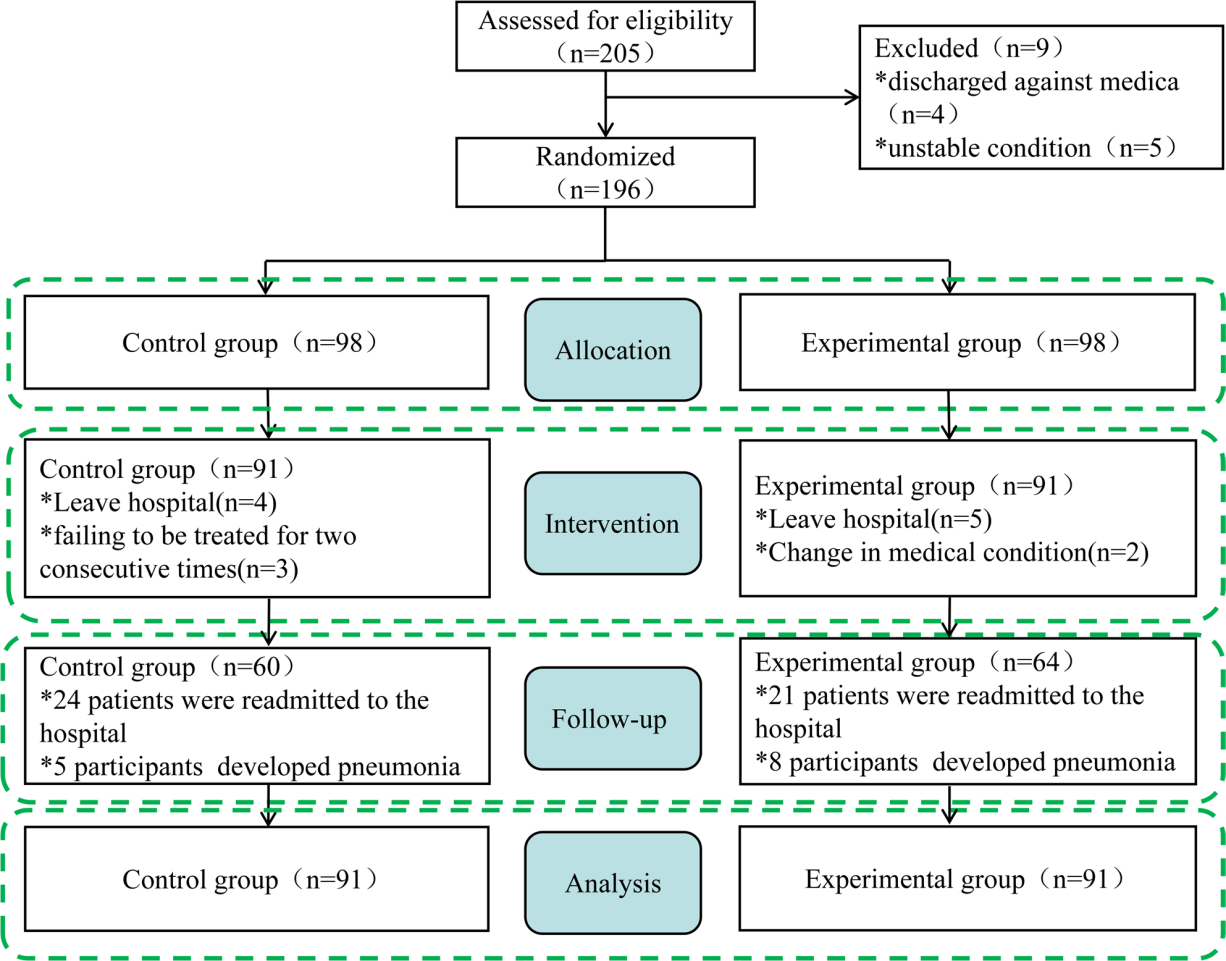
Flow diagram of the trial

This study was a randomized and controlled trial. 196 eligible participants were enrolled in this study. We followed the Consolidated Standards of Reporting Trials (CONSORT) statement. Decentralized randomization was conducted by an independent third party, blinded to the study participants’ characteristics, using a computer-generated random sequence combined with block randomization. No stratification took place. The block size was fixed at 4, with a 1:1 allocation ratio, meaning that within each block, two patients were assigned to the control group and two to the experimental group. Each patient recruited was registered and given the next sequential number within the nursing home, before the envelope was opened to determine their allocation. The third party then informed the independent rater (baseline measurements). Afterward, the therapists were notified to which group the study participant was assigned. Due to the intervention method, physical therapists were not blinded. Outcome assessments were conducted by neurorehabilitation physicians who were not involved in the intervention delivery and had no access to group allocation information. All procedures related to randomization, including sequence generation, allocation details, and supervisory information, were systematically documented. After the completion of all participant follow-ups and outcome assessments, the data were finalized and quality-checked, and an independent statistician performed the unblinding and conducted the statistical analyses. Figure 1 provides the flow diagram of patient screening, randomization, assessment, and intervention.

### Treatment methods

**Fig. 2:**
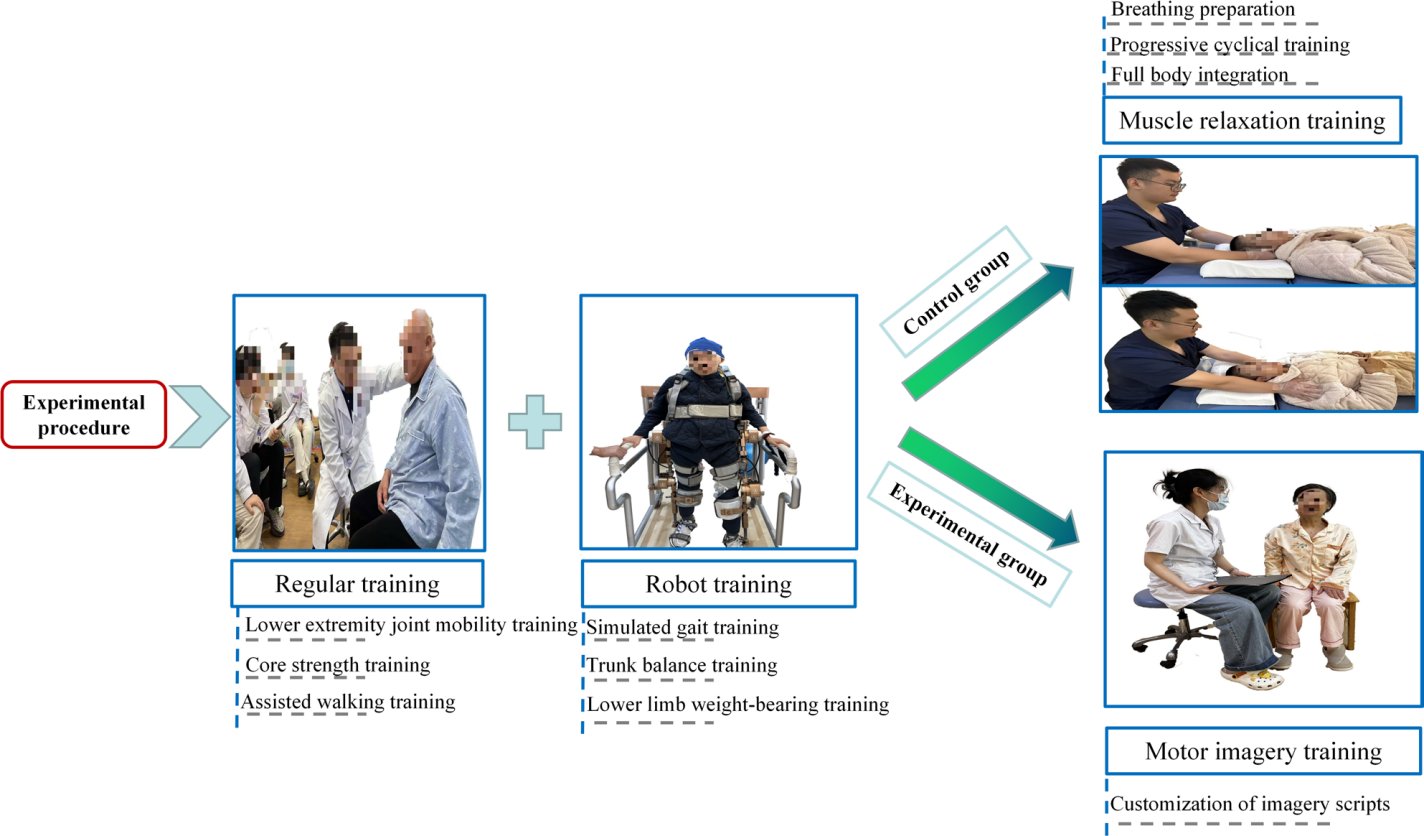
Study design and intervention flow

Both groups received conventional rehabilitation and task-oriented robot training, each session lasting 25 min. Subsequently, the experimental group received MI training, while the control group received muscle relaxation training, both interventions were administered for 20 min. Treatments were delivered once daily, five days per week, over four weeks, excluding weekends. The detailed intervention protocol is illustrated in Fig. 2.

#### Conventional rehabilitation treatment

Physical therapy includes routine lower extremity joint mobility training, core strength training, and assisted walking training.

#### Task-oriented robot training

Task-oriented robot training was conducted using the Beijing Daiai Lower Limb Robot (model AlWalker). Bilateral thigh and calf lengths were measured by the same therapist to ensure individualized adjustment. Patients were securely positioned in the device with a safety vest, and the weight-support system was activated to achieve upright standing. Handrails were used for upper limb support, and gait parameters were set as follows: initial step frequency of 35 steps/min, step length of 35–40 cm, and 100% guidance force. The hip-knee-ankle angle was set to gait mode 4 with an 80% scaling ratio.

The task-oriented protocol included three components: (1) Simulated gait training: Patients performed robot-assisted walking along a predefined path for 10 min per session; (2) Trunk balance training: With pelvic fixation in the robot, patients supported their upper limbs and trunk on a Bobath ball, performing guided anterior and lateral trunk movements for 10 min per session; (3) Lower limb weight-bearing training: The robot fixed the affected limb in mid-stance, and the therapist assisted with single-leg weight-bearing for 10 min per session.

#### MI training

In addition to the conventional rehabilitation treatment and task-oriented robot training, participants in the experimental group received MI training, which was adapted from the protocol developed by Oostra et al. [39]. Each participant received a 20-minute MI intervention daily, conducted in a quiet room by one experienced therapist who were not involved in outcome assessments. Sessions began with two minutes of relaxation to facilitate a focused mental state. Participants were seated comfortably with their eyes closed and were instructed to perform MI from an internal perspective, combining both visual (seeing oneself performing the action) and kinesthetic (feeling the movement) modalities, with emphasis placed on kinesthetic imagery.

During the first week, therapists familiarized participants with the principles of MI through multisensory cues, guiding them to imagine daily movement scenarios personally familiar to them. From the second week onward, training content was tailored to specific impairments observed in clinical gait analysis or lower limb motor assessments provided by physical therapists, such as joint stiffness, asymmetry, or impaired coordination. Imagery scripts were customized accordingly, focusing on critical movement components such as hip, knee, or ankle control for lower-limb rehabilitation, or grasping and reaching movements for upper-limb training.

During the third and fourth weeks, patients were guided to mentally rehearse integrated functional tasks, emphasizing temporal-spatial parameters such as step length, gait rhythm, or reaching accuracy. Where appropriate, auditory cues were provided to facilitate internal pacing. In the later phase, MI was embedded within meaningful activities of daily living, including walking across uneven terrain or reaching for household items, encouraging participants to mentally simulate task performance in a variety of real-life environments.

To maximize the therapeutic efficacy and maintain the internal validity of the MI intervention, a dynamic adjustment strategy based on the patient’s capacity was rigorously implemented. Patients’ initial imagery ability was quantified using the KVIQ-10 at baseline. This established whether a patient possessed a high or low imagery ability. Patients categorized with high ability received the standard intensity MI protocol, involving complex, multi-joint movement tasks, whereas began with a simplified MI protocol, focusing on single-joint movement tasks that were easier to clearly visualize and control.

Throughout the intervention period, therapists engaged patients in reflective dialogue, primarily using the weekly subjective ratings recorded in the adherence log (Likert scores for vividness and clarity), as quantitative feedback. This ongoing feedback was used to refine and adjust the imagery scripts and strategies. Crucially, the complexity of the low-ability group’s training tasks was dynamically adjusted and gradually increased based on their demonstrated improvement in subjective clarity and performance ratings, ensuring that the intervention remained both motivating and therapeutically aligned with each patient’s progress and needs.

#### Muscle relaxation training

The control group received an equal amount of muscle relaxation training in addition to conventional rehabilitation treatment and task-oriented robot training. Muscle relaxation training was implemented to ensure therapeutic attention, consisting of daily one-on-one sessions lasting 20 min each. This relaxation training adhered to the principles of progressive muscle relaxation as outlined by Salt and Kerr [41]. The fundamental concept behind this technique involves instructing participants to intentionally tense specific muscle groups in a predetermined sequence. The training consisted of three distinct phases: (1) Breathing preparation phase: The patient was positioned comfortably in a supine posture. The therapist guided them through abdominal breathing exercises designed to establish a foundational state of relaxation. (2) Progressive cyclical training phase: This core phase focused on the systematic “tensing-and-releasing” of twelve specific muscle groups, following a standardized sequence. The sequence included shoulder shrugging, forehead wrinkling, forceful eye closing, jaw clenching, abdominal tightening, back arching, bilateral knee extension and flexion, as well as ankle dorsi- and plantar-flexion, and toe flexion and extension. (3) Full body integration phase: During this final phase, the patient was guided to experience a sensation of limb heaviness and complete body relaxation while maintaining a steady breathing rhythm. Natural background soundscapes were employed to enhance and sustain a deeply relaxed state.

### Outcome evaluation

All assessments were conducted within one day before the first rehabilitation treatment and within one day after four weeks of treatment, with a total duration of approximately 1.5 h. Evaluations were completed by the neurorehabilitation physicians who did not know the specific subgroups and experimental methods of this study.

#### Motor imagery ability evaluation

The present study employed the Chinese version of the KVIQ-10 to assess participants’ MI ability. Originally developed by Malouin et al. [35], the KVIQ was designed to evaluate both visual imagery and kinesthetic imagery in individuals with physical disabilities and has demonstrated good reliability. The KVIQ-10 consists of 10 items, each comprising one visual and one kinesthetic sub-item, covering five movement categories: head rotation, shoulder elevation, trunk forward flexion, knee extension, and ankle dorsiflexion. All movements are performed in a seated position to accommodate individuals with balance or standing difficulties. The assessment follows a four-step procedure: (1) observation of the target movement, (2) physical execution of the movement, (3) MI practice with eyes closed (either visual or kinesthetic mode), and (4) subjective rating. A 5-point Likert scale is used for scoring: the visual imagery subscale evaluates the clarity of the imagined image (1 = no image at all, 5 = image as clear as actually seeing the movement), while the kinesthetic imagery subscale assesses the intensity of the movement sensation (1 = no sensation, 5 = sensation as vivid and real as actual movement). Higher scores indicate stronger imagery ability.

At the start of each MI training session, an adherence log was recorded by therapists to document patients’ experiences during the imagery sessions. This booklet included sections for recording details such as the session date, start time, duration, and ratings regarding their personal experience during performance, as well as the vividness and clarity of the images experienced during the imagery session. Responses were captured using 11-point Likert scales, with values ranging from − 5 (much weaker) to + 5 (much stronger) for the vividness and clarity.

#### Lower limb motor function evaluation

The motor function of the lower limbs in patients with stroke was assessed using the FMA-LE. Concurrently, additional outcomes, including balance function, activities of daily living, cortical activation, and brain network function, were also obtained.

Evaluation of lower limbs motor function, FMA-LE, is a clinically commonly used scale for assessing motor function in stroke patients, with good reliability and validity [42]. The maximum score of this 17-item scale is 34 points. Each item is scored on a 3-point ordinal scale, with 0 points for inability, 1 point for partial ability, and 2 points for full ability to perform the required movement. A higher score of FMA-LE means that patients have better lower limb motor function.

The balance function was assessed by the Berg Balance Scale (BBS), which is a highly validated scale used for evaluating balance in individuals with neurological conditions, exhibiting robust reliability and internal validity [43]. It achieves an intraclass correlation coefficient of 0.97 for inter-measure reliability and 0.98 for intra-measure reliability [44]. The BBS consists of 14 items, each rated on a scale ranging from 0 (indicating poor balance) to 4 (indicating excellent balance), resulting in a total score of 56 [45].

Gait spatio-temporal parameters (step frequency, step speed, step length, and gait cycle) were assessed using a video gait analysis system (Taiwan Longgu Wang Company, model: R019-5 A). The assessment was conducted in a room equipped with a specific 10-meter runway, ensuring the room remained sufficiently quiet. Patients wore tight-fitting pants or shorts and walked along the designated straight runway toward the motion capture camera from the starting point of the runway until the display screen completed the recording. During the walk, the assessment therapist protected the patient outside the runway to prevent falls. The test was conducted three times consecutively, and the average values of the corresponding parameters were taken.

#### NIRS data evaluation

The experiment was conducted in a quiet environment, with only the tester and participants present during the test. Firstly, participants were asked to sit quietly in comfortable seats to collect the eight-minute resting-state data, close their eyes, and avoid head movement. The assessment environment was standardized to ensure reliability: the room was quiet, clean, and well-lit, with a controlled temperature of 26–27 °C and humidity maintained at 55–60%.

#### Experimental equipment

**Fig. 3:**
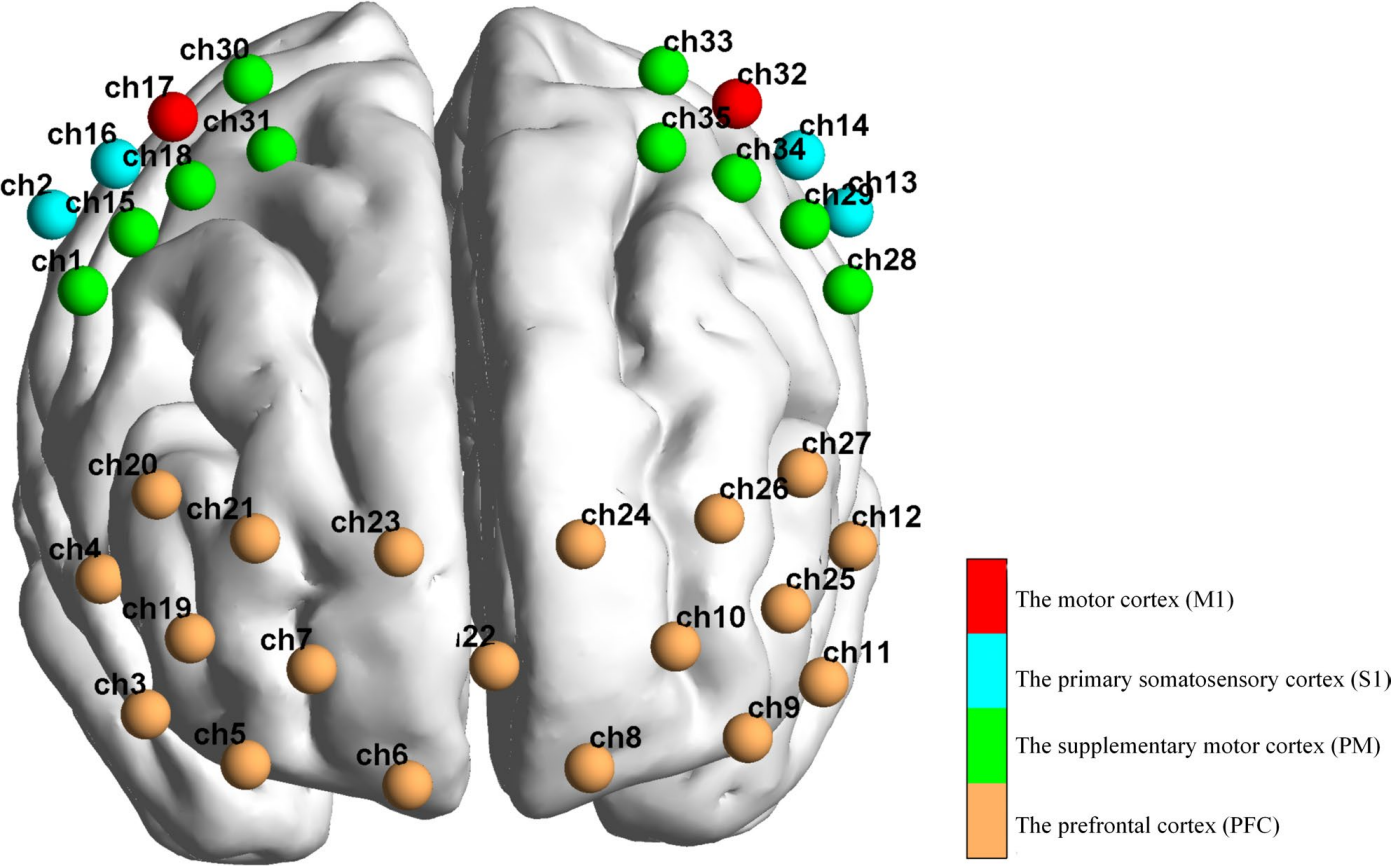
The schematic of the fNIRS channel locations on the head

A 35-channel fNIRS system with 14 sources and 14 detectors (NirSmart-3000B, Danyang Huichuang Medical Equipment Co. Ltd., China) was used to record changes in cerebral cortex oxygen concentrations. The distance between the headgear probe and the light source was 30 ​mm, and the brain area was below the midpoint of the channel (i.e., the midpoint of the connection between the emitter and the detector). The main detection area of this channel was positioned concerning the international 10–20 system, covering the forehead and both sides of the movement area. Near-infrared light signals at two wavelengths (730 ​nm and 850 ​nm) were continuously recorded, with a sampling frequency of 11 ​Hz. The channel brain area was calibrated regarding the Brodmann cerebral cortex partition. According to the coordinate information, the 35 channels were divided into 8 regions of interest (ROIs) in the left and right cerebral cortexes of the subject, including the left prefrontal cortex (LPFC, CH8, 9, 10, 11, 12, 24, 25, 26, 27), right prefrontal cortex (RPFC, CH3, 4, 5, 6, 7, 19, 20, 21, 23), left supplementary motor cortex (LPM, CH28, 29, 33, 34, 35), right supplementary motor cortex (RPM, CH1, 15, 18, 30, 31), left motor cortex (LM1, CH32), right motor cortex (RM1, CH17), left primary somatosensory cortex (LS1, CH13, 14), right primary somatosensory cortex (RS1, CH2, 16). Additionally, the primary motor area (M1) represents a cortical brain functional area related to lower limb movement (Fig. 3).

#### NIRS data processing

The MATLAB 2013b (MathWorks, Inc.; Natick, US) coupled with Homer2 was used to analyze the time series concentrations of HbO_2_ in the 8-minute resting-state data. Initially, we applied the hmrIntensity2OD function to convert the raw data into optical density values. Following this, a functional trimming method was used to remove channels with significantly low signal-to-noise ratios that diverged from the others. Motion artifacts were detected at the channel level using the hmrMotionArtifactByChannel function. Key detection parameters were set as follows: AMPthresh = 5.00, STDEVthresh = 50.0, tMotion = 0.5, and tMask = 1.0. We utilized a combined approach of Spline Interpolation hmrMotionCorrectSpline with *p* = 0.99 and turnon = 1, and Wavelet Transformation to repair the marked artifact segments. Our decision to combine spline interpolation with wavelet transformation was informed by Di Lorenzo’s [46] recommendation, which indicated that this combination provides optimal results for motion artifact correction while preserving the trial. In contrast to Di Lorenzo’s methodology, we opted for a more lenient interquartile range (iqr) value of 1.5 in the wavelet transformation to minimize the risk of losing critical information, ensuring that non-artifact data remained intact in the signal. Next, we utilized the stimulus suppression function to eliminate trials that commenced or concluded within 25.0 s before or 20.0 s after motion artifacts. Physiological noise was attenuated using a hmrBandpassFilt (hpf = 0.010, lpf = 0.10). Finally, the optical density values were converted into changes in hemoglobin concentration using the hmrOD2Conc function (ppf = 6.0, 6.0). The network module of ROIs was used to extract changes in HbO_2_ concentration at each time point, and the Pearson correlation coefficient of HbO_2_ concentration across channels in the time series was analyzed. After Fisher-*Z* transformation, the coefficient was defined as the functional connectivity between channels.

### Statistical analysis

Statistical analyses were conducted using SPSS 26.0 (IBM Corp., New York, USA). Visualization of results was performed with BrainNet Viewer and Origin 2021. Data normality was assessed using the Kolmogorov-Smirnov test. Homogeneity of variance test was used the Levene’s Test. The outcome measures, FMA-LE, BBS, gait spatio-temporal parameters, and the average of functional connectivity satisfied by both the normality and homogeneity of variance assumptions, and are presented as mean ± standard deviation. The categorical variables are reported as counts and percentages. Demographic characteristics were compared using independent samples t-tests or chi-square tests. Repeated Measures Analysis of Variance (ANOVA) was used to determine the interaction and main effects of FMA-LE, BBS, and gait spatio-temporal parameters. The difference of resting-state functional connectivity (rs-FC) in specific ROIs within groups was evaluated by paired-sample t-test, and the difference between groups in both before and after intervention was calculated by independent sample t-test of Δr (post-intervention subtract pre-intervention), with FDR correction for multiple comparisons. Partial correlation analysis was employed to examine associations between the FMA-LE scores and rs-FC after controlling for baseline data (age, stroke type, lesion side, and FMA-LE). Statistical significance was set at *p* < 0.05 (two-tailed).

## Results

### General information

In the control group, there were 65 males and 26 females, with an average age of 56.12 ± 10.44 years, an average disease course of 1.12 ± 0.95 months, and an average MMSE score of 27.03 ± 1.42. There were 50 hemorrhagic stroke and 41 ischemic stroke cases. There were 42 cases where the affected hemisphere was the left, while 49 cases were right. In the experimental group, there were 62 males and 29 females, with an average age of 56.92 ± 9.22 years, an average course of disease of 1.05 ± 0.70 months, and an average score of MMSE of 27.00 ± 1.64. There were 41 hemorrhagic stroke and 50 ischemic stroke cases. There were 54 cases where the affected hemisphere was the left, while 37 cases were right. There were no significant differences in gender, age, type of stroke, affected hemisphere, disease duration, MMSE, BBS, FMA-LE, Step frequency, Gait speed, Step length, Gait cycle, and KVIQ-10 scores between the two groups (*p* > 0.05). The baseline data have been outlined in Table 1.

**Table 1:** General information between the two groups

	Control group (*n* = 91)	Experimental group (*n* = 91)	T/χ^2^	*P*
Gender			0.235	0.628
Male	65 (71.4%)	62 (68.1%)		
Female	26 (28.6%)	29 (31.9%)		
Age (years)	56.12 ± 10.44	56.92 ± 9.22	-0.549	0.583
Stroke type			1.780	0.182
Hemorrhagic	50 (54.9%)	41 (45.1%)		
Ischemic	41 (45.1%)	50 (54.9%)		
Lesion side			3.174	0.075
Left	42 (46.2%)	54 (59.3%)		
Right	49 (53.8%)	37 (40.7%)		
Disease duration (months)	1.12 ± 0.95	1.05 ± 0.70	0.622	0.535
MMSE	27.03 ± 1.42	27.00 ± 1.64	0.145	0.885
BBS	38.54 ± 7.19	39.16 ± 7.81	-0.563	0.574
FMA-LE	21.68 ± 2.85	21.29 ± 2.98	0.915	0.361
Step frequency	1.00 ± 0.35	0.96 ± 0.31	0.805	0.422
Gait speed	0.31 ± 0.17	0.28 ± 0.14	1.233	0.219
Step length	0.34 ± 0.10	0.33 ± 0.09	1.005	0.316
Gait cycle	2.24 ± 0.79	2.27 ± 0.80	-0.259	0.796
KVIQ-10	33.74 ± 6.77	34.62 ± 8.37	-0.779	0.437

MMSE: Mini-Mental State Examination, BBS: Berg Balance Scale, FMA-LE: Fugl-Meyer Assessment – Lower Extremity, KVIQ-10: Kinesthetic and Visual Imagery Questionnaire – 10

### Comparison of motor imagery ability

**Fig. 4:**
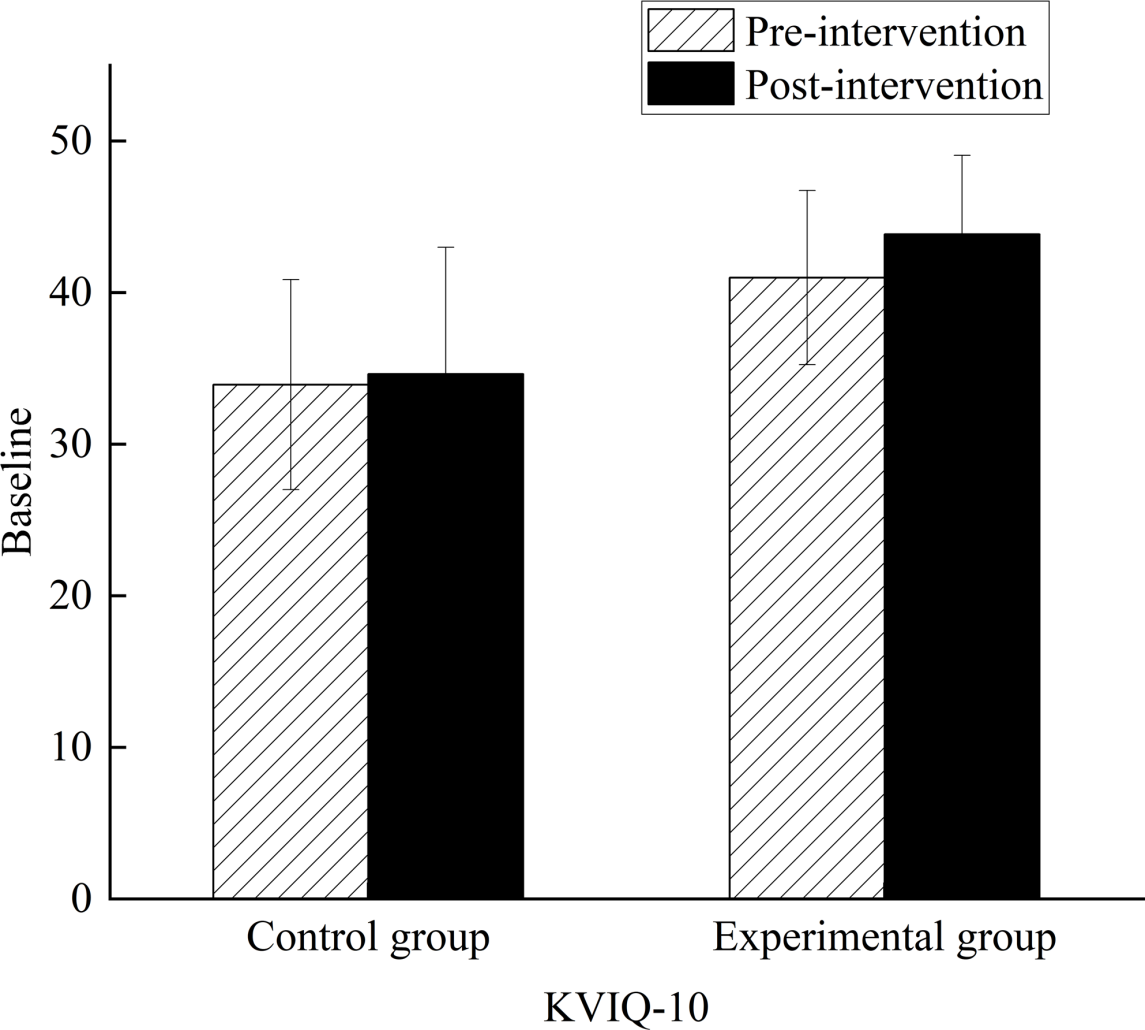
Comparison of MI ability scores

The log entries revealed that all participants completed an average of five MI training sessions per week (M = 19.40 ± 2.06 min), amounting to at least 20 sessions over the span of four weeks in the experimental group. The results indicated that a general increase in the vividness and clarity of their imagery across all participants, with measurements indicating improvements from the beginning to the end of the intervention session (MW1 = -19.47 ± 3.16; MW2 = -10.59 ± 2.02; MW3 = 10.68 ± 2.01; MW4 = 17.58 ± 3.52). Importantly, the results indicated that four weeks of MI training combined with lower limb robot training resulted in an increase of KVIQ-10 score compared with muscle relaxation training coupled with lower limb robot training (group × time interaction, F_1,180_ = 14.130; *p* < 0.001; η^2^_p_ = 0.073). Post hoc analysis revealed that the KVIQ-10 score increased in the control (*p* < 0.001; 95% CI: -7.858, -6.251) and experimental group (*p* < 0.001; 95% CI: -10.023, -8.416) after intervention compared with baseline. Post hoc analysis also revealed a significant difference between the two groups after the intervention (*p* < 0.001; 95% CI: -4.453, -1.240), whereas there was no significant difference between the two groups before the intervention (*p* = 0.550; 95% CI: -2.928, 1.565). Figure 4 shows the changes in MI ability, as represented in the bar chart.

### Comparison of lower limb motor function

The repeated ANOVA analysis results show that four weeks of MI training combined with lower limb robot training resulted in an increase of FMA-LE, BBS, Step frequency, and Gait speed compared with muscle relaxation training coupled with lower limb robot training (group × time interaction, all *p* < 0.05). Post hoc analysis revealed that FMA-LE, BBS, Step frequency, Gait speed, and Gait cycle score increased in the control (all *p* < 0.05) and experimental group (all *p* < 0.001) after intervention compared with baseline. Post hoc analysis also revealed a significant difference in FMA-LE and BB between the two groups after the intervention (all *p* < 0.05). In contrast, there was no significant difference between the two groups before the intervention (all *p* > 0.05). Table 2 shows the detailed results of motor function comparison of the two groups.

**Table 2:** Comparison of motor function scores

Variable/Group	Pre-intervention	Post-intervention	Main effect (Time) F(*p*)	Main effect (Group) F(*p*)	Interaction effect (Time*Group) F(*p*)
FMA-LE					
Control Group	21.68 ± 2.85	23.53 ± 3.22^a^	165.483 (<0.001)*	4.796 (0.03) *	27.467 (<0.001)*
Experimental Group	21.29 ± 2.98	25.67 ± 3.51^ab^			
BBS					
Control Group	38.54 ± 7.19	41.16 ± 5.94^a^	82.047 (<0.001)*	4.165 (0.043) *	8.049 (0.005)*
Experimental Group	39.16 ± 7.81	44.19 ± 5.48^ab^			
Step frequency					
Control Group	1.00 ± 0.35	1.18 ± 0.30^a^	147.8 (<0.001)*	0.001 (0.981)	5.18 (0.024)*
Experimental Group	0.96 ± 0.31	1.22 ± 0.26^a^			
Gait speed					
Control Group	0.31 ± 0.17	0.38 ± 0.15^a^	46.528 (<0.001)*	0.945 (0.332)	8.147 (0.005)*
Experimental Group	0.28 ± 0.14	0.44 ± 0.19^ab^			
Step length					
Control Group	0.35 ± 0.12	0.34 ± 0.10	0.114 (0.736)	2.332 (0.128)	0.138 (0.711)
Experimental Group	0.33 ± 0.11	0.33 ± 0.09			
Gait cycle					
Control Group	2.24 ± 0.79	1.77 ± 0.51^a^	130.214 (<0.001)*	0.02 (0.889)	0.177 (0.675)
Experimental Group	2.27 ± 0.80	1.76 ± 0.51^a^			

BBS: Berg Balance Scale, FMA-LE: Fugl-Meyer Assessment – Lower Extremity. “a” represents a significant difference of baseline (*p* < 0.05); “b” represents significantly different from the control group (*p* < 0.05). *Represents significant effect (*p* < 0.05)

To assess the stability of the intervention effect, post-hoc stratification analyses were performed. The three-way interaction for Stroke Type (Time * Group * Stroke Type) was not statistically significant (all *p* > 0.05), indicating that the treatment effect was consistent between ischemic and hemorrhagic stroke patients. Similarly, the interaction for Lesion Side (Time * Group * Lesion Side) was also non-significant (all *p* > 0.05), confirming that laterality did not modify the intervention response.

### Comparison of functional connectivity

**Fig. 5:**
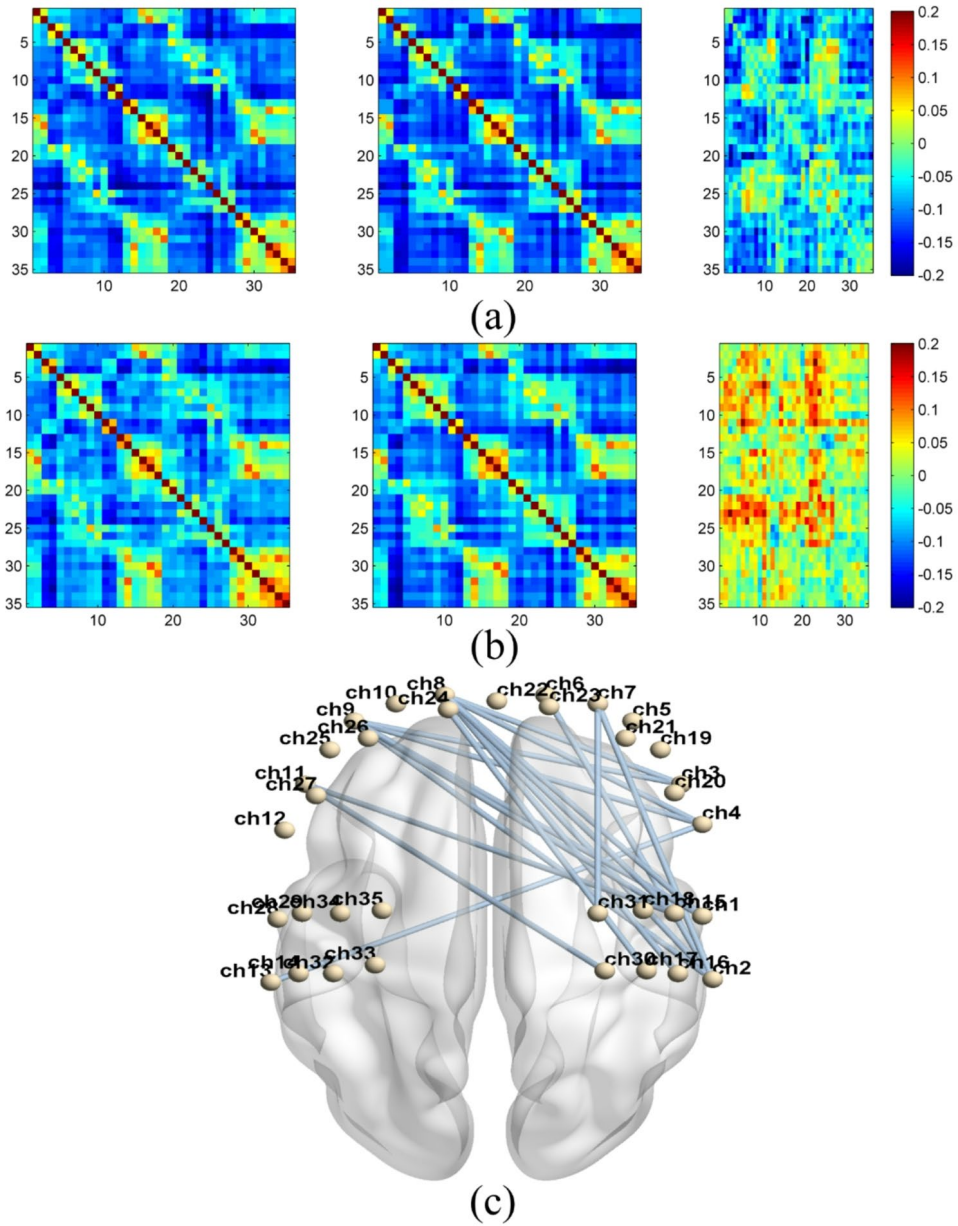
Comparision of functional connectivity. The functional connectivity of channels of control and experimental groups. **a** The mean and differences in the correlation matrix for the control group. **b** The means and differences in the correlation matrix for the experimental groups. For both (**a**) and (**b**), the left column represents the mean functional connectivity of channels at baseline, the middle column shows the mean functional connectivity of channels following the intervention, and the right column displays the differences in the correlation matrix. The color bar indicates Δr, where red signifies that the channels after intervention exhibited higher values than those at the baseline. **c** The figure depicts functional connectivity showing a statistically significant differential effect (Δr_experimental_ – Δr_control_). Δr represents the change in functional connectivity (post-intervention minus pre-intervention). Only connections significant after FDR correction are displayed

The functional connectivity across all brain regions was compiled into correlation matrices and analyzed within and between groups. Figure 5 illustrates the changes in rs-FC across all channels before and after the intervention.

The correlation matrix analysis conducted before and after the intervention indicates that the experimental group exhibited a greater improvement in channel functional connectivity post-intervention compared to the control group. As shown in Fig. 4(d), both groups demonstrated enhanced functional connectivity across channels. However, the experimental group showed a more significant increase in functional connectivity, including the PFC–motor cortex connectivity. The following significant increasing pairs were observed within the experimental group: LPFC-RPM (ch1-ch8, ch1-ch9, ch8-ch15, ch9-ch15, ch11-ch15, ch11-ch30), RPFC-RPM (ch7-ch31), RPFC-RS1 (ch2-ch6, ch2-ch7), LPFC-RS1 (ch2-ch8, ch2-ch9, ch8-ch16), LPFC-RPFC (ch3-ch8, ch3-ch9, ch4-ch8, ch4-ch9), RPFC-LS1 (ch4-ch13), and LPFC-RM1 (ch17-ch24) .

### Correlation of lower limb motor function and functional connectivity

To further confirm the association between changes in rs-FC increased by treatment, the Partial correlation analysis was conducted to analysis between the FMA-LE score and functional connectivity. We set the age, stroke type, lesion side, and FMA-LE baseline data as the control variables to control for the influence of baseline data on our findings. The results revealed a positive correlation between the FMA-LE scores and RM1-LPFC (ch17-ch24: *r*_*partial*_ (176) = 0.807, *p* < 0.001) and RM1-LPM (ch17-ch34: *r*_*partial*_ (176) = 0.233, *p* = 0.002) in patients after stroke.

### Follow up

At four months post-intervention, a telephone follow-up was conducted to evaluate the sustainability of lower limb functional recovery and enhancements in quality of life. A total of 124 participants were successfully contacted (Control group, *n* = 60; Experimental group, *n* = 64). However, 45 patients (Control group, *n* = 24; Experimental group, *n* = 21) were readmitted to the hospital for continued care due to high expectations for functional recovery and/or other functional impairments. Additionally, 13 participants (Control group, *n* = 5; Experimental group, *n* = 8) developed pneumonia. No instances of recurrent stroke or mortality were reported in either group.

The primary reasons for dropout included changes in or difficulty reaching patient contact information, health status deterioration that precluded participation, and withdrawal of consent by a small minority of participants. Follow-up results indicated that patients receiving a combination of MI and task-oriented robotic training generally sustained significant improvements in gait stability, balance, and activities of daily living (ADL) function.

## Discussion

This study utilized a meticulously designed RCT to examine the effects of combining MI with task-oriented robotic training on lower limb motor function and cortical functional reorganization in early-stage stroke patients. The principal findings supported our central hypothesis: compared to the control group, which received conventional rehabilitation alongside task-oriented robotic training alone, the experimental group that included additional MI training exhibited significantly greater enhancements in the FMA-LE and BBS scores. Notably, fNIRS data illustrated a distinct pattern of improved functional coupling post-intervention in the experimental group, especially between the prefrontal cortex and the motor-related cortex. Moreover, enhanced functional coupling was significantly positively correlated with improvements observed in FMA-LE scores. These results provide compelling evidence supporting a synergistic rehabilitation strategy that integrates peripheral mechanical assistance (task-oriented robotic training) with central neural modulation (PFC-MI), offering profound insights into the underlying neurophysiological mechanisms. However, the fNIRS findings of this study should be understood within a multimodal neuroimaging framework. Future study should integrate fNIRS with resting-state fMRI to examine whole-brain network co-changes and further elucidate the multilevel neural mechanisms underlying motor recovery after stroke.

Repeated measures ANOVA revealed that the experimental group exhibited significantly greater improvements in FMA-LE, BBS, step frequency, and gait speed compared to the control group. This finding robustly supports the potential of MI as an enhancer for task-oriented robotic training. Notably, the experimental group achieved over a 4-point higher FMA-LE score than the control group. While this aligns with previous studies on robotic training alone, it exceeds the 2.8-point mean improvement reported with standalone robotic interventions [47], underscoring the synergistic value of MI. The superior efficacy of combined MI and task-oriented robotic training compared to monotherapy may involve dual neural remodeling pathways. Robotic training, through task-specific repetitive inputs such as closed-loop hip-knee trajectory control provided by the exoskeleton robot and trunk balance tasks, strengthens spinal-cortical motor pattern generation by activating *Ia* afferent fibers [14], thereby facilitating motor cortex relearning of multi-joint coordination of the lower limbs [48]. Concurrently, MI training enhances the efficiency of cortical information transfer by customizing protocols to match suitable difficulty levels, thus optimizing the simulation of specific movement postures. This process predominantly engages central mechanisms, activating motor cortical networks overlapping with actual motor execution regions, including the premotor cortex, supplementary motor area, and primary sensorimotor cortex. Consequently, MI amplifies the peripheral sensory stimulation induced by task-oriented robotic training on central neural circuits, yielding synergistic effects that result in significant functional gains surpassing those of single therapies [16–18]. These findings provide crucial clinical evidence for optimizing early stroke rehabilitation strategies.

Conventional rehabilitation theories suggest motor function recovery relies solely on the reorganization of the primary M1 and corticospinal tract repair [49]. However, emerging evidence positions the PFC as a key hub for executive control and cognitive regulation, widely recognized as a core node mediating rehabilitation progression [20]. Consistent with this perspective, our fNIRS findings revealed enhanced functional coupling between the PFC and motor-related regions after combined MI and task-oriented robot training, especially in LPFC-RPM and LPFC-RS1 pairs, suggesting improved coordination within the executive control and motor network circuits. These results corroborate the critical role of PFC in correcting motor errors within cerebellar-basal ganglia circuits. Mechanistically, the PFC may enhance functional cooperation between cognitive control regions and motor execution areas, thereby improving information exchange efficiency [50] and rapid integration of error feedback. Furthermore, it may optimize corticospinal connectivity and improve the recruitment of spinal α-motoneurons through descending pathways [51], ultimately enhancing the transmission efficiency of motor commands elicited by task-oriented robotic training. These findings challenge Wolpaw’s “core motor network theory” and highlight the pivotal role of the executive control network in post-stroke compensating motor reorganization. Specifically, when corticospinal tract integrity is severely compromised, the PFC may facilitate a “cognitive-to-motor functional reorganization” through extrapyramidal pathways [52], proving new evidence that MI-induced neuroplasticity extends beyond primary motor circuits.

Another key finding was that changes in functional coupling between RM1 and LPFC (CH17-CH24) after intervention were positively correlated with improvements in stroke patients’ motor function scores. This result is consistent with previous research [53], indicating that MI training enhances intrinsic brain motor network coupling, thus accelerating motor function recovery. This effect may be attributed to our dual-modal MI protocol, which integrates both visual and kinesthetic strategies. Visual MI activates the dorsal visual stream and posterior parietal cortices, mediating the spatial representation of the motor scenario and the visualization of motor planning [54]. In contrast, kinesthetic MI engages the sensorimotor cortex, supplementary motor area, and premotor cortex, showing significant overlap with proprioceptive and motor execution networks [55]. This intentional dual-modal integration likely promotes neuroplasticity through parallel pathways and coexisting synergistic pathways, combining abstract spatial representation (visual) and the embodied motor feeling (kinesthetic) for a more complete movement simulation [56].

The most insightful finding is that MI enhances functional coupling and accelerates motor function recovery, suggesting a highly valuable and adaptable neurorehabilitation principle. Notably, the MI component itself is cost-neutral and easily scalable in diverse rehabilitation contexts. While robotic assistance provides high-intensity, measurable motor repetition training, the cognitive modulation (PFC involvement) driven by MI constitutes the key synergistic factor. However, the current intervention setup requires specialized, costly equipment, limiting its application to strictly controlled clinical environments. This restriction is a significant barrier to broader dissemination in rehabilitation settings. To improve the feasibility and scalability of this synergistic method, future research should focus on optimizing these simplified MI protocols and their integration with affordable repetitive-task practice, rather than relying on expensive robotic systems. This will help confirm the efficacy of this combined approach across a variety of rehabilitation contexts.

### Limitations and further study

This study has several limitations. First, fNIRS is restricted to measuring hemodynamic changes on the cortical surface, making it unable to assess deep subcortical structures like the basal ganglia and cerebellum, which are crucial for motor recovery. Moreover, fNIRS functional coupling reflects only the statistical co-variation of activity, not direct causal influence [57]. Crucially, the heterogeneity of stroke pathology (cortical or subcortical) likely contributes to the interindividual variability in prefrontal–motor connectivity. This is highlighted by findings on the necessity of parietal or temporal integrity for recovery in primate models [58] and the value of these models for studying subcortical stroke [59, 60]. Future studies should address these limitations by adopting multimodal neuroimaging approaches, integrating fNIRS with high-resolution MRI/CT lesion mapping [59, 61], to clarify the hierarchical and causal mechanisms underlying post-stroke neuroplasticity.

Second, therapists were not blinded to group allocation, which creates a potential performance bias [62]. Although independent, blinded outcome assessors minimized measurement bias, the therapists’ awareness could still influence intervention intensity and patient interactions. Future research should address this limitation by (1) providing standardization training to ensure therapists remain neutral regarding group allocation, and (2) employing an independent research assistant to randomly audit intervention session recordings for blinding integrity.

Third, an important limitation concerns the clinical heterogeneity of our sample. We did not stratify our analysis based on the precise location of the stroke lesion or the side of the lesion. Previous research suggests that different lesion types engage distinct compensatory networks, which significantly impact recovery patterns. This lack of stratification may introduce variability into our observed functional connectivity results and limit our conclusions about the influence of lesion characteristics on dual-modal intervention effectiveness. Future studies should focus on detailed CT/MRI lesion mapping for stratified analysis.

Finally, another limit is the short follow-up period. Primary outcomes were assessed immediately post-intervention and included only a brief telephone follow-up. Without a standardized, objective long-term functional assessment (for example, at 3 or 6 months), we cannot determine the sustained effectiveness of the combined intervention or the long-term stability of the observed PFC-Motor functional coupling changes. Future research should incorporate longer follow-up times for a clearer understanding of the therapy’s lasting impact on brain function and motor recovery.

## Conclusion

This study investigated the effects of combining MI with task-oriented robotic training on lower limb function in patients with subacute stroke. The results demonstrated that the combined intervention significantly improved lower limb motor function recovery and cortical functional reorganization in early stroke patients. Moreover, this study revealed that enhanced connectivity between the prefrontal cortex and motor-related regions is a possible neural mechanism underlying lower limb function recovery in stroke patients. These findings carry important clinical implications and offer valuable insights for clinicians engaged in the neurorehabilitation of stroke patients. Future research should incorporate multimodal neuroimaging and simplified MI-based interventions are warranted to further validate and translate this synergistic framework into broader clinical practice.

## Data Availability

The data that support the findings of this study are available from the corresponding author upon reasonable request.

## Abbreviations

ANOVA: Analysis of Variance
BBS: Berg Balance Scale
CONSORT: Consolidated Standards of Reporting Trials
CT: Computed Tomography
FMA-LE: Fugl-Meyer Assessment of Lower Extremity
fMRI: Functional Magnetic Resonance Imaging
fNIRS: Functional near-infrared spectroscopy
HbO_2_: Oxygenated hemoglobin
KVIQ-10: Kinesthetic and Visual Imagery Questionnaire-10
M1: Motor cortex
MI: Motor imagery
MMSE: Mini-Mental State Examination
MRI: Magnetic Resonance Imaging
PFC: Prefrontal cortex
PM: Supplementary motor cortex
rs-FC: Resting-state functional connectivity
S1: Primary somatosensory cortex
